# Bile acids and their receptors in hepatic immunity

**DOI:** 10.1016/j.livres.2025.01.005

**Published:** 2025-01-28

**Authors:** Stefano Fiorucci, Silvia Marchianò, Eleonora Distrutti, Michele Biagioli

**Affiliations:** a Department of Medicine and Surgery, University of Perugia, Perugia, Italy; b SC di Gastroenterologia ed Epatologia, Azienda Ospedaliera di Perugia, Perugia, Italy

**Keywords:** Bile acids (BAs), Immune regulation, Postbiotics, Gut microbiota, Receptors

## Abstract

Similarly to conventional steroids, bile acids function as signaling molecules, acting on a family of membrane and nuclear receptors. The best-characterized bile acid-regulated receptors are the farnesoid X receptor, activated by primary bile acids, and the G-protein-coupled bile acid receptor 1 (also known as Takeda G protein-coupled receptor 5), which is activated by secondary bile acids, such as lithocholic acid (LCA) and deoxycholic acid. Both the farnesoid X receptor and G-protein-coupled bile acid receptor 1 are expressed in cells of innate immunity, monocytes/macrophages, and natural killer cells. Their activation in these cells provides counter-regulatory signals that are inhibitory in nature and attenuate inflammation. In recent years, however, it has been increasingly appreciated that bile acids biotransformations by intestinal microbiota result in the formation of chemically different secondary bile acids that potently regulate adaptive immunity. The 3-oxoLCA and isoalloLCA, two LCA derivatives, bind receptors such as the retinoic acid receptor-related orphan receptor gamma t (RORγt) and the vitamin D receptor (VDR) that are expressed only by lymphoid cells, extending the regulatory role of bile acids to T cells, including T-helper 17 cells and type 3 innate lymphoid cells (ILC3). In this novel conceptual framework, bile acids have emerged as one of the main components of the postbiota, the waste array of chemical mediators generated by the intestinal microbiota. Deciphering the interaction of these mediators with the immune system in the intestine and liver is a novel and fascinating area of bile acid renaissance.

## 1 Introduction

Bile acids (BAs) are steroid molecules produced in the liver through cholesterol breakdown and play a vital role in mammalian biology. Beyond their primary functions in bile production and nutrient absorption, they act as signaling molecules by binding to various cell membranes and nuclear receptors, collectively known as BA-activated receptors (BARs).^1^^,^^2^ These receptors are widely distributed in the human body, with especially high levels found in the liver, gastrointestinal tract, brain, cardiovascular system, adipose tissues, endocrine glands, reproductive organs, and muscles.^1^^,^^3^ The two most extensively studied BARs family members, the farnesoid X receptor (FXR) and the G-protein-coupled BAR 1 (GPBAR1), also known as Takeda G protein-coupled receptor 5 (TGR5),^4^^,^^5^ are highly expressed not only in epithelial cells but also in neurons, glial cells, pancreatic beta-cells, L-cells, extracellular matrix cells, and immune cells.^6^ In immune cells, the activation of FXR and GPBAR1 triggers regulatory pathways that promote immune tolerance. In addition to the primary BAs synthesized in the liver, a large number of secondary BAs are produced in the intestine by the gut microbiota.^6^ Recent advances in mass spectrometry and computational tools, such as molecular networking and reverse metabolomics, have led to the discovery of a previously unappreciated variability of secondary BAs. In the last five years, over 200 new BAs have been identified.^7^ These findings, combined with insights from multi-omics techniques, spatial transcriptomics, single-cell analysis, and multiparametric flow cytometry, reveal that BAs and their derivatives are key mediators of the chemical interactions that link the gut microbiota and the host immune system.^7^ Thus, while the expression and function of FXR and GPBAR1 are restricted to innate immune cells of myeloid origin, recent studies have shown that secondary BAs might interact with lymphoid cells via receptors such as the vitamin D receptor (VDR) and the constitutive androstane receptor (CAR).^8^^,^^9^ Moreover, secondary BAs can function as receptor antagonists, inhibiting receptors such as the retinoic acid receptor-related orphan receptor gamma t (RORγt) expressed by T-helper 17 (Th17) cells and type 3 innate lymphoid cells (ILC3s), and leukemia inhibitory factor (LIF) receptor.^8^^,^^9^ This review explores the emerging concept of BAs as postbiotics in mediating the interaction of gut microbiota on liver and intestinal immune regulation and the potential for the development of novel therapies for immune-mediated liver diseases.

## 2 Synthesis and metabolism of BAs: implications for hepatic immunity

### 2.1 Hepatic BA synthesis and the role of the intestinal microbiota

**Fig. 1:**
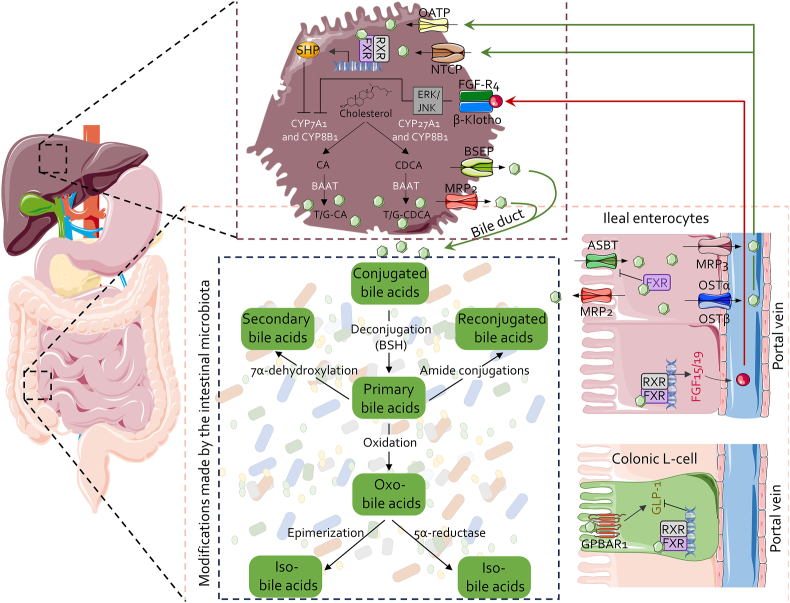
**Enterohepatic circulation.** Primary BAs, CA and CDCA, and their tauro- and glyco-conjugates, are synthesized in the liver from cholesterol via two metabolic pathways: the classical (or neutral) pathway, initiated by the enzymatic action of CYP7A1, leading to the synthesis of CA, and the alternative (or acidic) pathway, beginning with CYP27A1 activity and culminating in the synthesis of CDCA. BAs are transported into the bile ducts via the BSEP and MRP2 transporters. After a meal, BAs are released into the small intestine through the common bile duct, where they facilitate lipid absorption. In the gastrointestinal tract, BAs serve as substrates for numerous transformations catalyzed by gut microbiota, leading to the formation of secondary BAs, such as LCA and DCA, along with their derivatives. In the ileum, enterocytes absorb BAs via the ASBT transporter. BA entry into enterocytes activates FXR, which inhibits further BA reabsorption by blocking ASBT. FXR activation also induces the release of FGF15/19, which reaches the liver through the portal vein and inhibits BA synthesis via the FGF-R4/ERK/JNK pathway. BAs are then transported from enterocytes into the portal vein via the MRP3 and OSTα/β transporters, returning to the liver where they are reabsorbed by hepatocytes through OATP and NTCP. Elevated BA concentrations in hepatocytes inhibit BA synthesis by activating the FXR/SHP axis. In the colon, BAs interact with L-cells, where GPBAR1 promotes the release of GLP-1, while FXR exerts an opposing effect. Abbreviations: ASBT, apical sodium-dependent bile acid transporter; BAAT, bile acid-CoA: amino acid N-acyltransferase; BAs, bile acids; BSEP, bile salt export pump; BSH, bile salt hydrolases; CA, cholic acid; CDCA, chenodeoxycholic acid; CYP27A1, sterol 27-hydroxylase; CYP8B1, sterol 12α-hydroxylase; DCA, deoxycholic acid; ERK, extracellular signal-regulated kinases; FGF15/19, fibroblast growth factor 15/19; FXR, farnesoid X receptor; GLP-1, glucagon-like peptide-1; GPBAR1, G-protein-coupled bile acid receptor 1; JNK, c-Jun N-terminal kinases; LCA, lithocholic acid; MRP2, multidrug resistance associated protein 2; NTCP, sodium/bile acid cotransporter; OATPs, organic anion transporting polypeptides; OSTα/β, organic solute transporter alpha/beta; RXR, retinoid X receptors; SHP, small heterodimer partner.

BAs are hydroxylated steroids generated from cholesterol in the liver through a chain of reactions involving 17 distinct enzymes, the activity of which is regulated by FXR signaling. In hepatocytes, primary BAs (*e.g.,* cholic acid (CA) and chenodeoxycholic acid (CDCA)) are formed through two main metabolic pathways: the classical (neutral) pathway, initiated by cholesterol 7alpha-hydroxylase (CYP7A1, also known as cytochrome P450 7A1), or the alternative (acidic) pathway, driven by sterol 27-hydroxylase (CYP27A1). The rate of BA synthesis is regulated by FXR, a BA sensor, that modulates the expression and function of CYP7A1 via the small heterodimer partner (SHP), an atypical nuclear receptor that lacks the DNA binding domain. Furthermore, the gut microbiota and intestinal FXR might also regulate liver BA synthesis by regulating in addition to CYP7A1, the rate-limiting enzyme, also the CYP7B1, sterol 12α-hydroxylase (CYP8B1), and CYP27A1. These feedback regulations are partially mediated by fibroblast growth factor 19 (FGF19) (FGF15 is mouse ortholog), that is gut hormones that control homeostasis of BAs and glucose during the transition from the fed to the fasted state.^10^^,^^11^ This circuit regulates BA synthesis in the feeding-fasting transition (Fig. 1).

In hepatocytes, CA and CDCA are conjugated with glycine (75% in humans) or taurine (95% in mice); although recent discoveries have shown that a variety of amino acids, including phenylalanine, tyrosine or leucine, could be conjugated with BAs.^12^ These conjugation reactions, catalyzed by BA-coenzyme A (CoA) synthetase (BACS) and BA-CoA: amino acid N-acyltransferase (BAAT), reduce the pKa of BAs, increasing their solubility and facilitating their secretion into bile.^13^ Additionally, BAs can undergo conjugation with glucuronic acid by UDP-glucuronosyltransferases (UGT) or sulfation at positions C3, C7, and C27 by sulfotransferases (SULT); processes that, although minor under physiological conditions, become significantly upregulated in cholestatic disorders such as primary biliary cholangitis (PBC) and primary sclerosing cholangitis (PSC).^14^ Conjugated BAs are actively transported across the biliary membrane of hepatocytes by the bile salt export pump (BSEP/ABCB11) and multidrug resistance protein 3 (MDR3/ABCB4), initiating bile formation. Conjugated BAs are released from hepatocytes into the canalicular space, converge in the hepatic bile duct, and from the common bile duct into the gallbladder and/or ultimately to the duodenum, where they emulsify and promote the absorption of fats and fat-soluble nutrients.

In the distal ileum over 95% of BAs are reabsorbed and transported back to the liver via the portal circulation (entero-hepatic circulation), while a small proportion of BAs is excreted daily with the feces (∼0.2–0.6 g/day) (Fig. 1).^15^, ^16^, ^17^, ^18^ Within the intestine, particularly in the ileum and colon, primary BAs undergo a series of biotransformations operated by the gut microbiota, leading to the production of secondary BAs. Advanced metagenomic analyses and metabolomics have identified up to 97 distinct BA modifications, though this number is likely to grow with continued research. These biotransformations, categorized by enzymatic activities such as deamidation, 7-dehydroxylation, hydroxylation, oxidation, and epimerization, result in a wide variety of secondary BAs, some of which can re-enter hepatic circulation and modulate immune responses.^19^^,^^20^

Conjugated BAs firstly undergo deconjugation by removal of the glycine or taurine group. They are then transformed into secondary BAs, including deoxycholic acid (DCA) and lithocholic acid (LCA), as well as the BA isomers, including ursodeoxycholic acid (UDCA, ursodiol, the 7β isomer of CDCA, a tertiary BA formed in the liver by epimerization of the secondary BA LCA) and iso-deoxycholic acid (iso-DCA, 3β isomer of DCA), BA esters, and various unsaturated BA metabolites. These deconjugation steps are primarily mediated by bacterial bile salt hydrolase (BSH), which hydrolyzes the C-24 N-acyl bond, releasing free BAs.^16^ This process is particularly prominent in the ileum and proximal colon, regions densely populated by BSH-producing bacteria, including Gram-positive genera such as *Clostridium, Enterococcus, Bifidobacterium, Bacteroidetes,* and *Lactobacillus*.^21^, ^22^, ^23^ Deconjugated BAs were then de-hydroxylated by 7α-dehydroxylase, an enzyme that is mainly expressed by members of the *phylum Firmicutes* including genera Clostridium XIVa and XI, and *Clostridium scindens* and *C. hiranonis,* converting primary BAs into LCA and DCA.^24^ These modifications are significant because secondary BAs can act as either agonists or antagonists to various nuclear receptors, such as the RORγt, which plays a crucial role in hepatic immune regulation.^16^^,^^25^

The gene that encodes for 7α-dehydroxylase belongs to the family of BA-inducible (bai) genes encoding enzymes.^26^^,^^27^ The 7α-dehydroxylase is encoded by the bai operon, which is responsible for converting primary BAs into secondary BAs. The bai operon consists of 8 genes involved in different steps of the BA 7α-dehydroxylation process. Key genes in this operon include baiB, baiCD, baiE, baiA2, baiF, baiG, baiH, and baiI. Recently, the complete pathway for 7α-dehydroxylation was elucidated, with enzymes annotated for each step of the pathway. Monitoring the time-dependent conversion of CA to DCA in the presence of purified mixtures of Bai enzymes, it was shown that 6 enzymes out of 8 encoded by the bai operon are necessary and sufficient for the conversion *in vitro*. BaiB adds CoA to CA, which is then oxidized by BaiA2, forming 3-Oxo-cholyl-CoA.^15^ This intermediate is further oxidized to 3-Oxo-4,5-dehydrocholyl-CoA by BaiCD. BaiE then mediates the critical 7α-dehydroxylation to form 3-Oxo-4,5-6,7-didehydro-deoxy-cholyl-CoA. BaiH removes CoA from the intermediate, which is subsequently oxidized twice by BaiCD and BaiA2, respectively, to produce DCA. The baiG codes for transporter and is involved in the uptake of CA necessary for *in vivo* processes, while BaiI is dispensable in the 7α-dehydroxylation pathway of unconjugated BAs to secondary BAs. Similar dehydroxylation of CDCA leads to the formation of LCA. The BAI gene is typically found in anaerobic intestinal bacteria, particularly in species such as *Clostridium scindens*, *Clostridium hylemonae*, and other species within the *phylum Firmicutes*. One of the most well-studied bacteria involved in the 7α-dehydroxylation of primary BAs is the *Clostridium scindens*, which efficiently converts cholic acid into DCA and CDCA into LCA. *Clostridium hylemonae* is another prominent species capable of expressing the bai gene and contributing to secondary BA formation, including the production of LCA from CDCA. *Clostridium difficile*, while primarily known for causing antibiotic-associated diarrhea, also possesses some of the bai genes, though it does not metabolize BAs as efficiently as other species. Other species that express the bai gene are the *Eubacterium* species, such as Eubacterium sp. VPI 12708, which participates in BA metabolism. The *Ruminococcus* species: some species within the genus *Ruminococcus* also express bai genes and play a role in BA transformation.^28^

In addition to deconjugation and didehydroxylation, gut microbiota also mediates other modifications, including the oxidation and epimerization of BAs, which are mediated by hydroxysteroid dehydrogenase (HSDH). This catalyzes the oxidation/reduction of hydroxyl groups at the 3-, 7-, and 12-carbons of BAs,^21^ leading to the formation of UDCA and iso-DCA, -LCA, and -UDCA. These intermediates, which account for 20%–30% of BA metabolites produced in the colon, have gained attention for their ability to modulate immune responses by binding to receptors such as RORγt.^16^ Several HSDH species have been identified, including *Bacteroides, Clostridium, Eubacterium, Ruminococcus, Bifidobacterium, and Escherichia,* among others. The 12α- and 12β-HSDH have been shown to have great importance for their ability to generate 12-oxoLCA, a RORγt ligand.^29^ Additionally, *Odoribacter* and *Parabacteroides* expressing 3-oxo-5α-steroid 4-dehydrogenase bacteria generate -oxo-Δ4-LCA, 3-oxo-alloLCA, iso-alloLCA, and alloLCA, all of which function as RORγt and GPBAR1 ligands. Other less abundant BAs, such as hyocholic and hyodeoxycholic acids (HCA and HDCA), also contribute to the complex interactions between BAs and the immune system, particularly within hepatic environments.^25^

Beyond deconjugation, BAs may also undergo esterification with sugars, acetyl and methyl groups, or fatty acids. Some of these modified BAs, such as sulfates and glucuronidates, are predominantly excreted in human urine. The relative proportions of these BAs vary significantly under different physiological and pathological conditions, complicating the understanding of their roles in diseases such as cholestasis, obesity, and postbariatric surgery adaptation. The integration of metabolomics and lipidomics databases, including the Human Metabolome Database (HMDB, https://hmdb.ca/) and the LIPID Metabolites and Pathways Strategy (LIPID MAPS), has led to the identification of over 692 BA variants, encompassing 20 ring or core modifications and 110 carboxy tail modifications.^3^^,^^6^

#### 2.1.1 Desulfatation, esterification, and desaturation

BA sulfatase activity was found in the bacteria genera *Clostridium*, *Peptococcus*, *Fusobacterium*, and *Pseudomonas*. This sulfatase activity converts BA sulfates into less polar and more efficiently absorbed desulfated BAs. This desulfation results in the formation of more toxic BA with a longer half-life compared to its sulfated counterpart, which might be involved in hepatobiliary and intestinal toxicity, like cholestasis (a decrease in bile flow and biliary BA excretion) and colon cancers.^30^^,^^31^

#### 2.1.2 Microbial conjugated BAs

Primary BAs are conjugated with glycine and taurine at the C24 acyl site when secreted from the liver. However, a recent study has shown that in mice, BAs could be conjugated with various amino acids including phenylalanine, tyrosine, and leucine.^12^ Notably, phenyl-CA and tyrosine-CA were found to be human FXR agonists. Importantly, these conjugations were also detected in humans, particularly in patients with inflammatory bowel disease (IBD).^32^ The exact functions and reasons for these conjugates remain unknown, although the reconjugation may serve as a mechanism to reduce toxicity.^32^

### 2.2 Species-specific BAs and their impact on hepatic immunity

Rodents, particularly mice that are commonly used in experimental models, have a distinct BA profile compared to humans, primarily due to the presence of muricholic acids (MCAs), which are synthesized in the murine liver from CDCA by the action of cytochrome P450 family 2 subfamily C polypeptide 70 (Cyp2c70). The formation of MCAs involves a two-step enzymatic process: the initial 6β-hydroxylation of CDCA to form αMCA, followed by the epimerization of the C7 hydroxyl group to generate βMCA.^33^ In contrast to CDCA, which is a potent FXR agonist in humans, Tβ-MCA, one of the most abundant BAs in mice, is an FXR antagonist. This species-specific difference leads to a distinct BA pool in rodents, characterized by a more hydrophilic nature and potentially less cytotoxic effects compared to the human BA pool.^34^^,^^35^ Consequently, the physiological ligand for FXR in mice is CA, while in humans it is CDCA. Additionally, BAs in mice exert anti-FXR effects, significantly impacting BA synthesis and regulation.^1^

### 2.3 Bidirectional regulation between intestinal microbiota and BA metabolism

The composition of the intestinal microbiota significantly affects the size and composition of the BA pool, which, in turn, influences immune responses in the liver. Research on germ-free mice has demonstrated that the absence of microbiota leads to an expanded BA pool, with a particular accumulation of primary BAs such as Tα- and Tβ-MCA, which are strong FXR antagonists. This buildup suppresses intestinal FXR signaling and reduces the production of Fgf15, a critical regulator of BA synthesis in the liver. In contrast, in conventional mice with a normal microbiome, the presence of gut microbiota regulates BA production through FXR-dependent pathways, emphasizing the two-way regulation between the microbiota and liver BA metabolism.^36^

Modifying the gut microbiota through diet or probiotics can also affect this regulatory balance. For instance, giving mice the antioxidant tempol or the probiotic VSL#3 has been found to raise Tβ-MCA levels, thereby inhibiting FXR/Fgf15 signaling and increasing BA production in the liver.^37^ On the other hand, BAs themselves have antimicrobial properties that shape gut microbial communities, promoting bile-tolerant species while suppressing bile-sensitive ones. This interaction between BAs and the gut microbiota is crucial for maintaining intestinal balance and preventing conditions like bacterial overgrowth, which can negatively affect liver immunity. Clinical studies have shown that treatment with the FXR agonist obeticholic acid (OCA) alters the composition of the human gut microbiota, increasing the number of beneficial Gram-positive strains like *Streptococcus thermophilus* and *Lactobacillus* species while reducing pathogenic bacteria.^38^^,^^39^

### 2.4 Enterohepatic circulation

The enterohepatic circulation plays a critical role in the recycling of BAs, which is essential for the maintenance of the BA pool and for supporting hepatic immune functions. In humans, the composition of BAs varies significantly across different tissues and fluids. For example, CA, DCA, and CDCA are predominantly unconjugated in the blood, while conjugated BAs are more prevalent in the gallbladder and ileum. In contrast, the colon and feces primarily contain unconjugated DCA. Approximately 95% of BAs reaching the terminal ileum are reabsorbed and transported back to the liver via the portal vein, completing the enterohepatic circulation (Fig. 1). This highly integrated process ensures the conservation and recycling of BA species, maintaining the stability of the BA pool, which is crucial not only for nutrient absorption but also for the regulation of hepatic immunity. Within hepatocytes, reabsorbed BAs play a pivotal role in regulating the expression of enzymes such as CYP7A1 and CYP8B1, thereby integrating *de novo* BA synthesis with the existing BA pool. This regulation is vital for maintaining hepatic immune homeostasis and preventing immune-mediated liver diseases.^40^^,^^41^

## 3 BAs as receptor ligands and their role in hepatic immunity

**Fig. 2:**
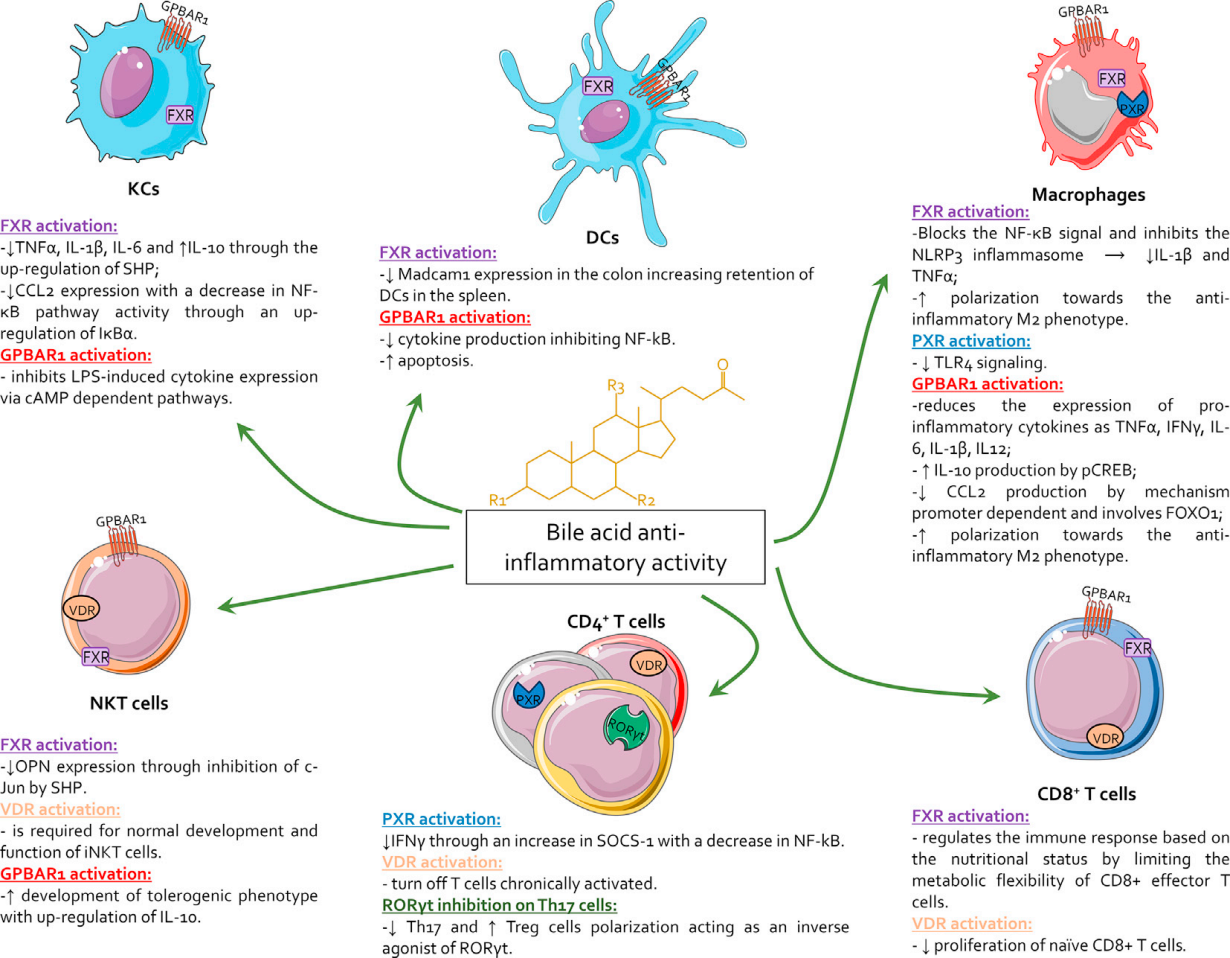
**Activation of bile acid receptors across different immune cell types.** FXR activation inhibits proinflammatory signaling pathways (*e.g.,* NF-κB and NLRP3 inflammasome) and promotes anti-inflammatory polarization of macrophages toward the M2 phenotype, while reducing cytokine production in KCs and DCs. GPBAR1 activation decreases the expression of proinflammatory cytokines, including TNF α, IFN γ, and IL-6, and enhances IL-10 production, contributing to M2 macrophage polarization and the inhibition of LPS-induced inflammatory responses. VDR and PXR activations further modulate immune responses by controlling CD8^+^ and CD4^+^ T cell activity, downregulating Th17 polarization, and promoting Treg differentiation. Together, these signaling pathways highlight the complex interplay between bile acid metabolism and immune regulation within the liver and gastrointestinal tract. Moreover, bile acids act as inverse agonists of RORγt, which is expressed on CD4^+^ T cells, inhibiting their polarization towards the Th17 subtype and promoting their differentiation into Treg cells. Abbreviations: cAMP, cyclic adenosine monophosphate; CCL2, chemokine (C-C motif) ligand 2; CD, cluster of differentiation; DCs, dendritic cells; FOXO1, forkhead box protein O1; FXR, farnesoid X receptor; GPBAR1, G protein-coupled bile acid receptor 1; IFNγ, interferon gamma; IL, interleukin; iNKT, invariant natural killer T; KCs, kupffer cells; LPS, lipopolysaccharide; NLRP3, NOD-like receptor family pyrin domain containing 3; NF-κB, nuclear factor kappa-light-chain-enhancer of activated B cells; OPN, osteopontin; pCREB, phosphorylation of the cAMP response element binding protein; PXR, pregnane X receptor; RORγt, retinoic acid receptor-related orphan receptor gamma t; SHP, small heterodimer partner; SOCS-1, suppressor of cytokine signaling-1; TNF α, tumor necrosis factor alpha; TLR4, Toll-like receptor 4; Treg cells, regulatory T cells; Th cells, T helper cells; VDR, vitamin D receptor.

BAs, as steroidal molecules, serve as endogenous ligands for a variety of cell membrane and nuclear receptors, playing crucial roles in regulating immune and inflammatory responses. This section delves deeply into the roles of two key BARs—GPBAR1 and FXR—in hepatic immunity, highlighting their mechanisms of action, physiological significance, and potential therapeutic implications (Fig. 2).

### 3.1 GPBAR1 (TGR5)

GPBAR1 is a G protein-coupled receptor primarily activated by secondary BAs such as DCA and LCA, as well as their taurine (T) and glycine (G) conjugates. Discovered in 2002 by Maruyama *et al.*,^42^ GPBAR1 is extensively expressed in several tissues relevant to hepatic and intestinal physiology. Its expression is most pronounced in the ileum and colon, particularly in epithelial cells, endocrine cells, and neurons of the intestinal tract. In the liver, GPBAR1 is found in non-parenchymal cells, including liver sinusoidal endothelial cells (LSECs), Kupffer cells (KCs), and hepatic stellate cells (HSCs), but is notably absent in hepatocytes.^42^

GPBAR1 expression is also significant in various immune cells, particularly those involved in innate immunity, such as monocytes, macrophages, dendritic cells (DCs), and natural killer T (NKT) cells. The receptor’s presence in these cells underscores its importance in modulating immune responses, particularly in the context of inflammation and tissue homeostasis within the liver and intestines.^2^^,^^5^^,^^43^

GPBAR1 plays a crucial role in dampening inflammatory responses,^44^ particularly through the inhibition of nuclear factor kappa-light-chain-enhancer of activated B cells (NF-κB) signaling and the suppression of inflammasome assembly.^43^^,^^45^ NF-κB is a central transcription factor that regulates the expression of various proinflammatory cytokines and is activated in response to numerous stimuli, including infections, tissue injury, and stress.^46^ Upon activation by its ligands, GPBAR1 initiates a signaling cascade involving the activation of adenylyl cyclase (AC), leading to increased levels of cyclic adenosine monophosphate (cAMP).^47^ This, in turn, activates protein kinase A (PKA), which phosphorylates key target proteins involved in the regulation of inflammation. One critical pathway involves the phosphorylation of cAMP response element-binding protein (CREB), which then translocates to the nucleus and binds to CRE sites in the promoters of anti-inflammatory genes, such as interleukin (IL)-10.^48^ This results in the upregulation of IL-10, a potent anti-inflammatory cytokine that plays a pivotal role in limiting immune responses and preventing excessive inflammation.^49^

Moreover, GPBAR1 directly inhibits NF-κB signaling by preventing the phosphorylation and degradation of IκBα, an inhibitor of NF-κB. Specifically, GPBAR1 activation promotes the binding of β-catenin to IκBα, thereby protecting it from IκB kinase (IKK)-mediated phosphorylation and subsequent degradation. This mechanism effectively prevents the nuclear translocation of the NF-κB p65/RelA subunit, thereby reducing the transcription of proinflammatory genes such as TNF-α and IL-1β.^45^

In addition to its effects on NF-κB, GPBAR1 is reported to modulate the assembly of the NOD-like receptor family pyrin domain containing 3 (NLRP3) inflammasome, a multiprotein complex that responds to a variety of danger signals, including pathogen-associated molecular patterns (PAMPs) and damage-associated molecular patterns (DAMPs).^50^ GPBAR1 activation leads to the phosphorylation of NLRP3 at Ser295 (in humans) or Ser291 (in mice) via a cAMP-PKA-dependent pathway. This phosphorylation event promotes the ubiquitination of NLRP3, preventing its assembly into the inflammasome complex and thereby reducing the production of proinflammatory cytokines such as IL-1β and IL-18.^51^^,^^52^

The role of GPBAR1 in hepatic immunity extends to its protective effects in various liver diseases. GPBAR1 deficiency has been shown to exacerbate inflammation and tissue damage in models of cholestasis, metabolic dysfunction-associated steatohepatitis (MASH), and liver fibrosis. For instance, in cholestatic liver disease, the absence of GPBAR1 leads to increased infiltration of inflammatory cells, enhanced activation of HSCs, and subsequent fibrosis.^53^ Conversely, activation of GPBAR1 with natural or synthetic agonists has been demonstrated to mitigate these effects, highlighting the receptor’s potential as a therapeutic target in liver diseases.^54^

The therapeutic potential of GPBAR1 is further underscored by its ability to modulate immune cell trafficking and cytokine production in the liver. By inhibiting the recruitment of leukocytes and reducing the production of proinflammatory mediators, GPBAR1 agonists can protect against liver injury and promote tissue repair. Additionally, the flexible binding site of GPBAR1 allows for the development of hybrid molecules that can target multiple receptors, further expanding the therapeutic applications of GPBAR1 agonists in treating inflammatory liver conditions.^55^

### 3.2 FXR in immunity

The FXR, a nuclear receptor was identified in 1995,^56^ and then deorphanized in 1999.^4^^,^^57^^,^^58^ Animal study has shown that FXR plays an important role in various metabolic processes, including BA homeostasis, lipid and glucose metabolism, and immune responses.^2^ In humans, FXR is predominantly activated by CDCA, although it can also respond to other BAs and their derivatives. FXR is highly expressed in the liver and intestine, particularly in hepatocytes, enterocytes, and cholangiocytes, where it plays a critical role in maintaining metabolic and immune balance.^56^, ^57^, ^58^

In the immune system, FXR expression is largely restricted to myeloid cells,^59^ including monocytes, macrophages, DCs, and natural killer (NK) cells.^60^^,^^61^ Its expression in T cells is minimal, suggesting a more specialized role in modulating innate immune responses at the epithelial interface, rather than adaptive immunity.^3^^,^^6^^,^^62^

FXR is the main BA sensor in mammalians and controls the expression of genes involved in BA synthesis, transport, and detoxification.^4^^,^^58^ Upon activation by BAs, FXR forms a heterodimer with the retinoid X receptor (RXR) and binds to specific DNA sequences known as FXR response elements in the promoters of target genes.^58^ This binding induces the expression of key regulatory proteins, such as the small heterodimer partner (SHP) and FGF19 (Fgf15 in mice), which together suppress BA synthesis in the liver and promote BA excretion into the bile.^36^^,^^63^

In addition to its role in BA metabolism, FXR also regulates lipid homeostasis by modulating the expression of genes involved in cholesterol and triglyceride metabolism. FXR activation leads to the suppression of lipogenic genes and the induction of genes involved in fatty acid oxidation, thereby reducing hepatic fat accumulation and preventing the development of steatosis and MASH.^64^

FXR exerts significant immunomodulatory effects, particularly in the context of hepatic inflammation and fibrosis. These effects are mediated through both SHP-dependent and SHP-independent mechanisms.^60^ SHP, an atypical nuclear receptor that lacks a DNA-binding domain, acts as a transcriptional corepressor and is a key mediator of FXR’s anti-inflammatory actions.^65^ Upon FXR activation, SHP represses the expression of proinflammatory cytokines such as IL-1β and tumor necrosis factor-α (TNF-α) by inhibiting the activity of transcription factors like c-Jun and NF-κB.^66^, ^67^, ^68^

In addition to SHP, FXR also interacts with nuclear corepressor 1 (NCoR1) to regulate the expression of inflammatory genes. FXR activation stabilizes NCoR1 on the promoters of these genes, preventing NF-κB from binding and thereby down-regulating the expression of inflammatory mediators such as inducible nitric oxide synthase (iNOS) and IL-1β. This mechanism is particularly relevant in the context of liver inflammation, where FXR activation can protect against endotoxin-induced liver injury and sepsis.^69^^,^^70^

Additionally, it has been shown that FXR regulates inflammasome activity, particularly the NLRP3 inflammasome. In liver macrophages, FXR activation inhibits NLRP3 assembly, thereby reducing the production of proinflammatory cytokines and mitigating the inflammatory response to bacterial endotoxins. Studies in animal models have shown that FXR agonists can protect against the development of shock and severe liver injury in response to bacterial infection, highlighting the receptor's potential as a therapeutic target in inflammatory liver diseases.^71^

The role of FXR in liver disease is multifaceted, encompassing the regulation of BA and lipid homeostasis and immune responses. FXR agonists have shown promise in the treatment of a variety of liver conditions, including PBC,^72^ PSC, and metabolic dysfunction-associated steatotic liver disease (MASLD).^72^ By reducing BA toxicity, suppressing hepatic inflammation, and preventing fibrosis, FXR agonists offer a comprehensive approach to managing these chronic liver diseases.^73^^,^^74^

The development of FXR agonists has advanced rapidly in recent years, with several compounds currently in clinical trials.^75^ These agonists are designed to selectively activate FXR in the liver and intestines, minimizing off-target effects and enhancing therapeutic efficacy. Interestingly, even FXR antagonists have proven effective in modulating intestinal and liver immunity, with beneficial effects in cholestasis.^76^ The ongoing research in FXR signaling pathways continues to reveal new opportunities for targeting this receptor in the treatment of liver and metabolic diseases, positioning FXR as a central player in hepatic immunity and disease management.^77^

### 3.3 Other membrane receptors

#### 3.3.1 S1PR2

The sphingosine-1-phosphate receptor 2 (S1PR2) is another G protein-coupled receptor that interacts with BAs, particularly taurocholic acid and taurochenodeoxycholic acid. S1PR2 is expressed in the liver and modulates various physiological processes, including lipid metabolism, immune cell trafficking, and inflammatory responses.^78^^,^^79^ Activation of S1PR2 in immune cells generally enhances phagocytosis, but can also promote inflammation depending on the context, such as in acute liver injury or cholestasis.

#### 3.3.2 LIFR

The leukemia inhibitory factor receptor (LIFR), part of the type I cytokine receptor family, mediates the effects of the leukemia inhibitory factor (LIF) on immune cells. LIFR activation triggers downstream signaling pathways, including the JAK/STAT pathway, which is involved in various immune responses and neoplasia.^80^, ^81^, ^82^ Recent studies have identified BAs, particularly DCA and LCA, as LIFR antagonists, suggesting a potential role in modulating inflammation and tumor progression in hepatic and gastrointestinal contexts.^83^^,^^84^

### 3.4 Other nuclear receptors

#### 3.4.1 PXR

The pregnane X receptor (PXR) is a nuclear receptor that plays a central role in the regulation of xenobiotic metabolism. It is primarily activated by BAs such as LCA, leading to the induction of detoxification enzymes and transporters that protect the liver from BA-induced toxicity.^85^, ^86^, ^87^, ^88^ Recently, PXR has gained attention for its significant role in modulating immune responses, particularly within the liver, where its anti-inflammatory effects can greatly influence liver immunity and disease progression.^89^

##### 3.4.1.1 PXR and inflammatory response regulation

One of the primary ways PXR exerts its immunomodulatory effects is through the regulation of inflammatory pathways. Specifically, PXR activation suppresses the expression of key proinflammatory cytokines such as TNF-α, IL-1β, and IL-6. These cytokines are crucial mediators of liver inflammation, and their reduced transcription following PXR activation helps attenuate the inflammatory response triggered by liver injury.^90^

A major mechanism underlying PXR’s anti-inflammatory action involves its crosstalk with the NF-κB, since PXR activation inhibits NF-κB signaling, thereby preventing the exacerbation of inflammation.^89^ This interaction might have relevance in managing liver inflammation in conditions such as cholestatic liver diseases, where PXR agonists have found utility in managing pruritus in PBC and PSC patients.^73^

KCs, the liver’s resident macrophages, are key mediators of the liver’s immune response.^91^ Upon liver injury, they produce proinflammatory cytokines, further promoting inflammation. However, PXR activation can suppress KC activity, leading to a reduction in the release of these inflammatory mediators. The modulation of KCs by PXR is critical in maintaining the balance between necessary immune defense and the prevention of chronic liver inflammation, which can otherwise result in long-term liver damage.

PXR also influences adaptive immune responses, particularly through its effects on T cells.^92^ While this area is less studied, evidence suggests that PXR activation can reduce T cell infiltration into the liver and suppress the activation of proinflammatory T cell subsets such as Th1 and Th17. These subsets are associated with exacerbating liver inflammation in various diseases. Moreover, PXR may also enhance the function of regulatory T cells (Tregs), which are known to suppress inflammation, further contributing to an immunosuppressive and tolerogenic environment within the liver.^92^

PXR is also expressed in HSCs.^93^ These cells are responsible for the production of extracellular matrix components and become activated during liver injury, contributing to fibrosis. PXR plays an indirect but significant role in modulating HSC activation. By reducing proinflammatory signaling, PXR limits the stimuli that drive HSCs into an activated state, thereby mitigating the fibrotic response. This capacity to suppress fibrosis through immune modulation highlights PXR’s potential as a therapeutic target in chronic liver diseases characterized by fibrosis.

For all these reasons, PXR’s immunomodulatory effects are particularly relevant in several liver diseases. In MASH, PXR activation has been shown to reduce liver inflammation by inhibiting proinflammatory pathways. Studies suggest that PXR agonists could serve as therapeutic agents in managing MASH by preventing immune cell infiltration and dampening inflammatory responses.^94^

In drug-induced liver injury (DILI), the effect of PXR activation in enhancing xenobiotic metabolism and suppressing immune activation helps protect the liver from immune-mediated damage. Similarly, in autoimmune liver diseases, where the immune system mistakenly targets liver cells, PXR activation offers a protective mechanism, though further research is needed to fully understand its potential in these conditions.^2^

In conclusion, PXR serves as a crucial regulator of liver immunity by attenuating proinflammatory signaling, particularly through interactions with pathways such as NF-κB. Its ability to modulate the activity of KCs, adaptive immune cells, and HSCs underscores its importance in controlling liver inflammation and fibrosis. By influencing both innate and adaptive immune responses, PXR maintains liver homeostasis and may offer a therapeutic avenue in managing inflammatory and fibrotic liver diseases such as MASH, DILI, and autoimmune liver disorders.^91^

##### 3.4.1.2 Impact on the gut-liver axis

In addition to its role in the liver, PXR is also expressed in the gut, where it contributes to maintaining intestinal barrier integrity and modulating the gut microbiome. By strengthening the gut barrier, PXR reduces the translocation of endotoxins, such as bacterial lipopolysaccharide (LPS), from the gut into the liver. This prevents excessive activation of immune cells, including KCs, thereby protecting the liver from inflammation driven by gut-derived toxins. The ability of PXR to influence the gut-liver axis adds another layer of immune protection, particularly in conditions where gut permeability and microbiome composition play roles in liver disease.^95^, ^96^, ^97^, ^98^

#### 3.4.2 VDR

The VDR is a nuclear receptor that mediates the immunoregulatory effects of vitamin D, beyond its traditional role in calcium and phosphate metabolism. While its primary ligand is 1α, 25-dihydroxycholecalciferol, VDR can also be activated by BAs such as LCA, although this interaction is relatively weak.^9^^,^^99^^,^^100^ It is thought that the immunomodulatory effects observed in the intestine and liver may also involve other receptors like GPBAR1 and RORγt.^101^, ^102^, ^103^ VDR expression in various immune cells and liver tissues highlights its significant role in regulating both innate and adaptive immune responses, which are critical for liver immunity and the management of liver-related diseases.

##### 3.4.2.1 VDR and immune responses

VDR activation significantly modulates the activity of KCs by reducing the production of proinflammatory cytokines, with an effect similar to that exerted by PXR activation. Furthermore, VDR regulates Toll-like receptor 4 (TLR4) expression, a receptor that detects PAMPs like bacterial LPS. By inhibiting TLR4 activation, VDR reduces KC activation, thereby limiting the downstream inflammatory response. Additionally, DCs are influenced by VDR activation. VDR promotes a tolerogenic phenotype in DCs, reducing their ability to trigger proinflammatory T cell responses. Consequently, this shift favors the development of Tregs, which suppress inflammation and contribute to immune tolerance in the liver.^104^

Moreover, VDR is expressed in various T cell subsets and plays a crucial role in modulating their activity. Activation of VDR suppresses the differentiation and function of Th1 and Th17 cells, which are major drivers of inflammation in the liver. Th1 cells produce interferon-γ (IFN-γ), and Th17 cells secrete IL-17, which both contribute to liver inflammation and injury. In contrast, VDR enhances the development and function of Tregs, which are essential for maintaining immune tolerance.^8^^,^^9^^,^^105^ These cells help prevent excessive immune activation and inflammation, thereby protecting the liver from immune-mediated damage.

VDR also modulates B cell activity, inhibiting their activation and the production of antibodies that may contribute to liver damage, particularly in autoimmune liver diseases. This modulation of B cells is important in controlling the adaptive immune response in conditions such as autoimmune hepatitis (AIH).

Furthermore, VDR signaling plays a protective role by inhibiting the activation of HSCs. Vitamin D, through VDR, prevents the transformation of quiescent HSCs into myofibroblasts, which are the main collagen-producing cells during fibrosis. Additionally, VDR reduces the production of profibrotic factors like TGF-β. VDR activation also exhibits antioxidant properties, reducing oxidative stress in the liver—a key driver of HSC activation and fibrosis progression. By limiting oxidative stress, VDR helps to curtail the fibrotic processes that can lead to chronic liver scarring.^106^

VDR plays a critical role in protecting against autoimmune liver diseases by regulating immune tolerance and modulating both innate and adaptive immune responses. In AIH, VDR enhances the function of Tregs, which suppress autoreactive immune responses, thereby reducing liver damage. In PBC, VDR modulates immune activity, reducing inflammation and slowing disease progression.^107^ Additionally, VDR is crucial in liver regeneration and repair, particularly following injuries from viral hepatitis or toxic damage caused by alcohol or drugs. Its ability to regulate immune responses limits liver damage reduces fibrosis by inhibiting HSC activation, and promotes tissue healing. Overall, VDR’s combined anti-inflammatory, antifibrotic, and regenerative effects make it a vital factor in maintaining liver homeostasis and preventing the progression of liver diseases.^108^^,^^109^

##### 3.4.2.2 VDR and the gut-liver axis

VDR plays a critical role in maintaining gut barrier integrity, which is essential for preventing the translocation of microbial products, such as LPS, from the intestine to the liver. A compromised gut barrier is often observed in liver diseases, where microbial products can enter the liver through the portal circulation, triggering immune activation. VDR activation strengthens gut barrier function by promoting the maintenance of tight junction proteins in the intestinal epithelium, thereby reducing bacterial translocation and subsequent immune activation in the liver.^3^

Moreover, VDR can modulate the composition of the gut microbiota, which is crucial for controlling inflammation. Dysbiosis, or an imbalance in the gut microbiota, is commonly associated with liver diseases like MASLD and alcoholic liver disease.^110^ By maintaining a healthy gut microbiome, VDR indirectly protects the liver from immune-driven inflammation.

#### 3.4.3 CAR

The CAR is a nuclear receptor primarily recognized for its role in xenobiotic and drug metabolism, similar to the PXR. However, beyond this function, CAR plays a significant role in modulating liver immunity, influencing both innate and adaptive immune responses, and contributing to the progression or mitigation of liver diseases such as MASH, DILI, and liver fibrosis.^90^

##### 3.4.3.1 CAR and immune responses

CAR activation affects the liver’s innate immune system, particularly through its modulation of KCs. Similar to PXR, the activation of CAR reduces the production of proinflammatory cytokines and suppresses the activity of the NF-κB signaling pathway, thereby limiting immune cell recruitment and inflammation in conditions such as MASH, DILI, or intestinal inflammation.^111^ Moreover, CAR can regulate TLR signaling, specifically TLR4. In terms of adaptive immunity, CAR impacts T cell function by reducing the recruitment and activation of proinflammatory T cells, particularly Th1 and Th17 cells linked to liver inflammation and fibrosis. At the same time, CAR can indirectly enhance the development of Tregs, which suppress excessive immune responses and help maintain immune tolerance, protecting against autoimmune reactions, such as those observed in AIH.

One of CAR’s critical functions is the inhibition of HSC activation. Activated HSCs produce extracellular matrix components that lead to fibrosis, and CAR suppresses both proinflammatory and profibrotic signals, including TGF-β. By inhibiting genes responsible for collagen production and fibrogenic pathways, such as matrix metalloproteinase activity, CAR limits the formation of scar tissue, reducing the fibrotic burden in chronic liver diseases like MASH.^112^

CAR is also crucial in protecting against DILI. It enhances the liver’s detoxification capacity by upregulating cytochrome P450 enzymes, notably CYP2B6 and CYP3A4, which are involved in metabolizing xenobiotics and clearing toxic substances.^113^ Furthermore, CAR modulates immune responses in DILI by reducing immune cell recruitment to sites of liver injury and suppressing the release of DAMPs, which can trigger further immune activation.

Furthermore, CAR interacts with other nuclear receptors such as PXR and FXR, coordinating responses to metabolic and immune challenges in the liver. While CAR and PXR share some functions in drug metabolism and immune regulation, their roles differ in the context of certain xenobiotics. FXR, which is involved in BA metabolism and anti-inflammatory responses, works alongside CAR to reduce liver inflammation, particularly in conditions such as cholestasis and MASH.^114^

##### 3.4.3.2 CAR and the gut-liver axis

Moreover, CAR influences the gut-liver axis by regulating BA metabolism and affecting the gut microbiome. A balanced gut microbiota is essential for preventing liver inflammation due to bacterial translocation and endotoxemia. CAR activation helps maintain gut microbiota balance, reducing the influx of microbial products and the resulting immune activation in the liver.^115^

#### 3.4.4 RORγt

RORγt is a nuclear receptor critical for the differentiation of Th17 cells and the regulation of intestinal immunity.^108^ BA derivatives, including oxo- and iso-forms of LCA and DCA, function as inverse agonists for RORγt, inhibiting Th17 cell differentiation and promoting Treg responses.^116^ These interactions highlight the complex role of BAs in balancing immune responses in the gut and liver.^44^^,^^105^^,^^116^, ^117^, ^118^

Th17 cells produce proinflammatory cytokines, particularly IL-17, which play a crucial role in intestinal immune responses. However, when dysregulated, they can contribute to inflammatory conditions such as IBD.^119^ There is evidence that isoalloLCA and 3-oxoLCA attenuate intestinal inflammation by inhibiting RORγt, thereby reducing the number of Th17 cells and consequently the production of inflammatory cytokines and chemokines.^32^^,^^120^, ^121^, ^122^ RORγt also drives the function of ILC3s, which are important for mucosal defense and maintaining barrier function in the gut.^9^^,^^123^ ILC3s produce IL-22, a cytokine that helps to control bacterial populations and promote tissue repair in the intestine. By modulating RORγt activity, BAs influence the function of ILC3s, indirectly controlling mucosal immunity and ensuring the maintenance of an appropriate balance between protective immunity and tolerance to commensal bacteria, which is crucial for preventing gut inflammation and maintaining overall gut health. By inhibiting RORγt, BAs also alter the gut microbial environment by promoting bile-tolerant species. This microbial modulation affects the composition of microbial metabolites that reach the liver via the portal vein, influencing the liver’s immune responses. By promoting regulatory immune pathways in the gut, RORγt-modulating BAs contribute to liver immune tolerance, preventing overactivation of immune cells in the liver.^6^ This might have clinical relevance since dysregulation of the BA-RORγt axis can lead to immune imbalances, which may contribute to liver diseases like MASLD and MASH. Importantly, this pathway might have potential therapeutic implications in these conditions.^121^

While RORγt is more directly involved in the regulation of intestinal immunity, its effects can extend to the liver through immune and metabolic crosstalk between the gut and liver. Indeed, by reducing Th17 cell activity and proinflammatory cytokine production in the intestine, RORγt helps to prevent excessive immune activation in the liver. Of relevance, RORγt interacts with Tregs, which are critical for maintaining immune tolerance. Tregs suppress inflammatory responses and promote tolerance to self-antigens, which is crucial for preventing immune-mediated liver damage.^124^ A balance between Th17 cells and Tregs is essential for immune homeostasis. Since the gut and liver are closely connected, disturbances in gut immunity can directly impact liver immunity. RORγt-dependent regulation of ILC3s helps maintain gut barrier function, preventing the translocation of bacteria and microbial products into the liver, which could trigger liver inflammation.^125^

## 4 BARs and hepatic immune regulation

### 4.1 Liver immune cells in metabolic syndrome and MASH

MASLD represents a spectrum of highly prevalent liver disorders characterized by the excessive accumulation of lipids within hepatocytes (hepatic steatosis) in individuals who have not significantly consumed alcohol or been exposed to hepatotoxic drugs.^74^ MASLD is histologically categorized into simple steatosis, referred to as metabolic-associated steatosis, and the more severe form, MASH. While metabolic-associated steatosis is characterized by hepatic steatosis without signs of hepatocellular injury, MASH is defined by the presence of steatosis accompanied by inflammation, hepatocyte injury (such as ballooning), and varying degrees of fibrosis. Unlike metabolic-associated steatosis, MASH is a progressive disease and has become the leading cause of chronic liver disease worldwide, primarily driven by the global rise in diabetes and obesity. Over the next 10–15 years, the prevalence of MASH is expected to increase, with a growing number of patients developing fibrosis—a critical determinant of both liver-related and overall mortality.^126^, ^127^, ^128^

Worldwide, the development of MASLD is strongly associated with sedentary lifestyles and dietary habits, and is closely linked to metabolic syndrome (including insulin resistance, increased body mass index, hypertension, and dyslipidemia) as well as its clinical manifestations such as obesity and type 2 diabetes mellitus.^74^ For these reasons, the reclassification of MASLD to MASLD emphasizes the metabolic underpinnings of this condition. However, while this reclassification provides a clearer understanding of MASLD as the hepatic manifestation of metabolic syndrome, it does not fully account for cases where MASLD develops in the absence of obesity, known as lean MASLD. Despite this limitation, the shift towards a metabolic-centered definition highlights the importance of developing therapeutic strategies needed to address this condition.^74^^,^^126^

MASH, which is associated with mild insulin resistance,^74^ can typically be managed with lifestyle modifications and is not currently considered a prime target for pharmacological intervention. However, when these lifestyle changes fail to achieve or sustain therapeutic benefits, this indicates a significant unmet clinical need. In such cases, similar to the management of type 2 diabetes, pharmacological treatments should be considered.^129^ Treatment strategies for MASH patients are primarily informed by the presence and severity of metabolic syndrome and the staging of liver fibrosis.^93^^,^^130^ At present, only resmetirom,^131^ a thyroid mimetic, has been approved by the U.S. Food and Drug Administration (FDA) specifically for the treatment of MASH, although various glucagon-like peptide 1 (GLP1) and glucose-dependent insulinotropic polypeptide (GIP) single, dual, or triple agonists are rapidly advancing toward approval.^132^^,^^133^ Various pathogenic mechanisms have been identified in MASH, and animal studies have demonstrated that disease progression or reversal can be achieved through diverse biochemical pathways. However, in clinical practice, the primary goal of treatment is to prevent the progression to cirrhosis, reduce the necessity for liver transplantation, and improve overall survival.^134^ Given that liver fibrosis, in addition to metabolic syndrome, is the main predictor of prognosis in MASH patients, medical treatments are particularly indicated for those with clinically significant fibrosis. However, liver fibrosis is a slowly progressing condition, and the development of cirrhosis and its complications can take decades, making the drug discovery and development process for MASH extremely challenging.

**Fig. 3:**
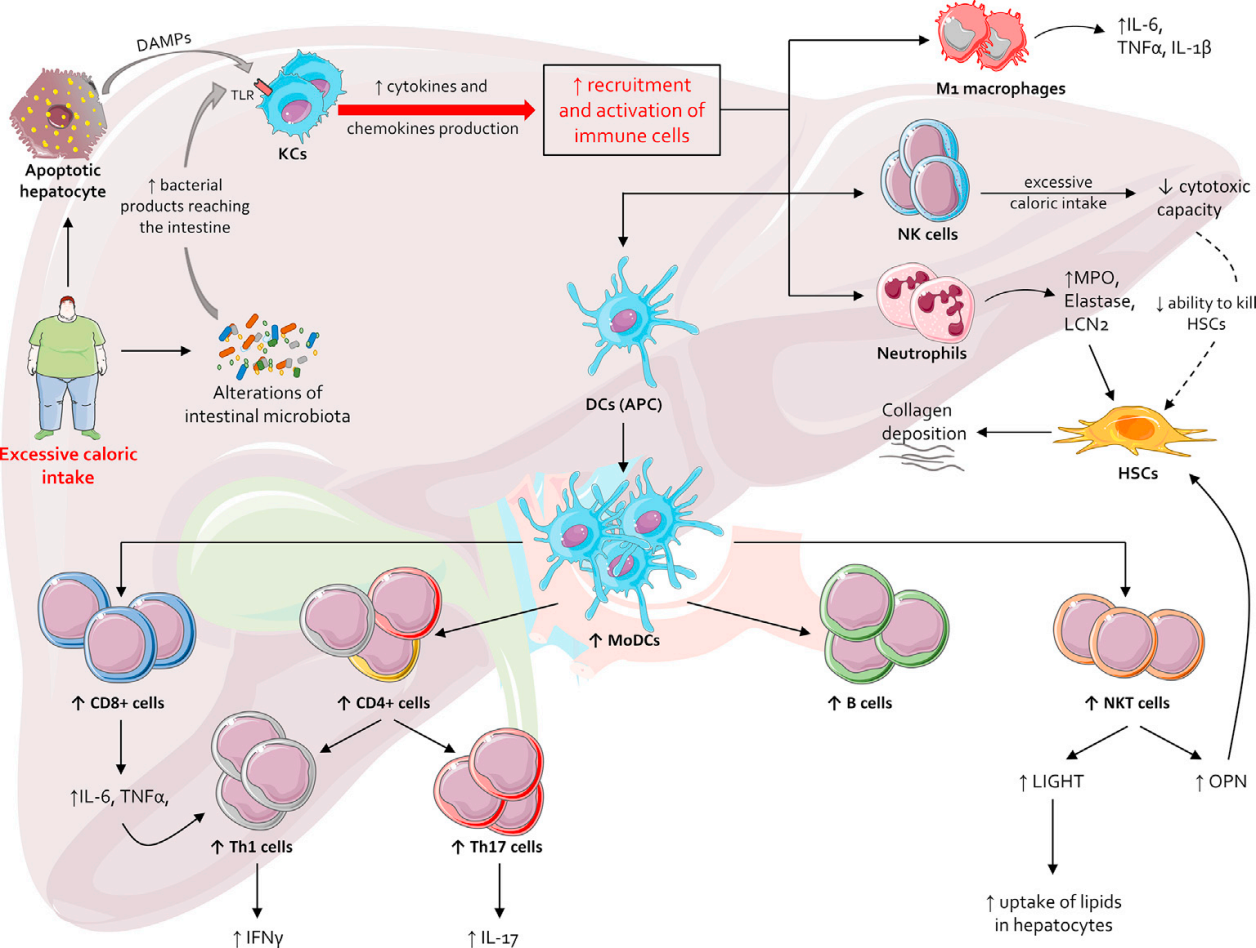
**Immune activation and fibrosis in MAFLD/MASH.** Excessive lipid intake leads to two primary pathological processes. First, it promotes hepatic lipid accumulation, triggering apoptosis and the release of DAMPs. Second, it increases intestinal permeability, facilitating the translocation of bacteria and bacterial components from the intestinal lumen into the circulation and subsequently to the liver. Both DAMPs and bacterial products activate KCs through TLRs. KC activation is the pivotal event that initiates the innate immune response, followed by the adaptive immune response. Activated KCs secrete proinflammatory cytokines (*e.g.,* IL-6, IL-1β, and TNF α), which upregulate chemokines, leading to immune cell recruitment in the liver. Neutrophils infiltrating the liver release high levels of MPO, elastase, and LCN2, further enhancing chemokine expression and HSC activation. Circulating macrophages, stimulated by liver-derived proinflammatory signals, polarize towards an M1 phenotype, producing additional proinflammatory cytokines. In MASLD, excessive caloric intake impairs the cytotoxic function of NK cells, reducing their ability to eliminate activated HSCs, thus promoting collagen deposition. DCs recruited to the liver act as APCs, driving the activation of the adaptive immune system. In MASLD, there is increased recruitment of DCs in hepatic sinusoids, while in MASH, hepatic NKT cells are elevated, producing OPN that activates HSCs, leading to collagen deposition, and LIGHT, which enhances hepatic lipid uptake, exacerbating steatosis. APCs also recruit CD4^+^ T cells, which preferentially differentiate into Th1 and Th17 subtypes, producing IFN γ and IL-17, respectively. Furthermore, MASLD is associated with an increase in CD8^+^ T cells and B lymphocytes in the liver. Abbreviations: APC, antigen-presenting cell; DAMPs, damage-associated molecular patterns; DCs, dendritic cells; HSCs, hepatic stellate cells; IFNγ, interferon γ; IL, interleukin; LCN2, lipocalin 2; LIGHT, lymphotoxin-like inducible protein; MAFLD, metabolic-associated fatty liver disease; MASLD, metabolic dysfunction-associated steatotic liver disease; MASH, metabolic dysfunction-associated steatohepatitis; MoDCs, monocyte-derived DCs; MPO, myeloperoxidase; NK cells, natural killer cells; NKT cells, natural killer T cells; OPN, osteopontin; Th cells, T helper cells; TLRs, Toll-like receptors; TNFα, tumor necrosis factor α.

Immune cells are central to the inflammatory response and fibrogenesis in MASLD (Fig. 3). Innate immune cells (*e.g.,* KCs, macrophages, neutrophils, NK cells) initiate inflammation and recruit adaptive immune cells (*e.g.,* T cells, B cells), which sustain and amplify the damage to hepatocytes and HSCs, promoting liver fibrosis. The crosstalk between immune cells, hepatocytes, and the gut-liver axis is essential in driving the disease’s progression.^93^^,^^135^

Immune cells of both innate and adaptive immunity participate in MASLD development.^93^^,^^136^^,^^137^

#### 4.1.1 Innate immune cells

• KCs (liver-resident macrophages): KCs are activated in the early stages of MASH due to the accumulation of free fatty acids, lipotoxicity, and gut-derived endotoxins (such as LPS).^138^ Once activated, they release proinflammatory cytokines (*e.g.,* TNF-α, IL-1β, and IL-6) that contribute to liver inflammation, recruit more immune cells, and induce hepatocyte damage.
• Monocyte-derived macrophages: These macrophages are recruited to the liver during MASH. They release proinflammatory cytokines and chemokines, contributing to chronic inflammation and promoting fibrosis by activating HSCs, the key cells responsible for liver fibrosis.^135^^,^^139^^,^^140^
• Neutrophils: These cells are recruited to the liver and release reactive oxygen species and proteases, which exacerbate hepatocyte injury and inflammation in MASH.^2^
• DCs: They process and present antigens to T cells, initiating adaptive immune responses. In MASH, DCs are activated by DAMPs and can contribute to both inflammation and fibrogenesis.^141^
• NK and NKT cells: NK and NKT cells have dual roles in MASH.^128^^,^^142^ While NK cells can help control fibrosis by killing activated HSCs, both NK and NKT cells also contribute to the inflammatory environment, worsening liver damage.

#### 4.1.2 Adhesion molecules

The family of cell adhesion molecules (CAMs) includes four major groups: selectins, integrins, cadherins, and members of the immunoglobulin superfamily of CAMs. In addition, non-classical CAMs such as vascular adhesion protein 1 (VAP-1), mucosal addressin CAM-1 (MAdCAM-1), and stabilins play a role in regulating liver immune responses.^143^ CAMs play a central role in regulating leukocyte trafficking toward the liver and have been targeted pharmacologically in several pathological processes, including fibrosis and cholestasis.^144^ MAdCAM-1, which binds to α4β1, α4β7, and L-selectin, is expressed almost exclusively by endothelial cells in the gastrointestinal tract and by the gut-associated lymphoid tissue. However, in PSC, MAdCAM-1 expression is induced on liver sinusoidal cells in the portal tract and is thought to make a contribution in recruiting leukocytes from the portal circulation providing a link between intestinal inflammation and PSC.^145^^,^^146^ Various BARs, including FXR, have been shown effective in reducing MAdCAM expression in the intestine, by a mechanism that could be mediated by inhibition of TNFα/NF-κB signaling.^146^ In addition to FXR, GPBAR1 has been shown to modulate the expression of various integrins such as vascular cell adhesion molecule (VCAM) and intercellular adhesion molecule on LSECs, HSCs, and platelet/endothelial cell adhesion molecule-1.^48^^,^^147^ Part of these effects is mediated by inhibition of NF-κB, but might also involve the release of gaseous mediators such as nitric oxide or hydrogen sulfide.^148^^,^^149^

#### 4.1.3 Adaptive immune cells

• T cells: In MASH, several types of T cells contribute to liver injury:^150^, ^151^, ^152^
  ○ CD4^+^ Th cells: Th1 and Th17 cells are particularly involved in MASH progression. Th1 cells release proinflammatory cytokines like IFN-γ, while Th17 cells release IL-17, both of which contribute to liver inflammation and fibrosis.
  ○ CD8^+^ cytotoxic T cells: These cells directly kill hepatocytes and contribute to liver injury. Their activation is associated with the progression of liver inflammation in MASH.^2^
• Tregs: Tregs play a protective role by suppressing inflammation. However, in MASH, their numbers or function may be impaired, leading to unchecked inflammation.^152^
• B cells: B cells, though less studied in MASH, are involved in adaptive immunity. They may contribute by producing antibodies that promote immune responses or by presenting antigens to T cells.

#### 4.1.4 Crosstalk between immune cells and metabolic cells

• Hepatocytes: Damaged hepatocytes release DAMPs, which trigger the activation of immune cells like macrophages and DCs, amplifying the immune response. Hepatocytes also produce chemokines that recruit immune cells to the liver.^140^
• HSCs: HSCs are activated by cytokines and chemokines released by immune cells, leading to their transformation into myofibroblasts, which produce extracellular matrix and drive fibrosis. Immune cells, especially macrophages, play a key role in activating HSCs.^153^^,^^154^
• Adipocytes: In obesity, adipocytes release proinflammatory adipokines (*e.g.,* TNF-α, IL-6) and free fatty acids that promote liver inflammation and immune cell activation, linking metabolic dysfunction with liver pathology in MASH.^155^

#### 4.1.5 Gut-liver axis

The gut microbiota also plays a role in MASH progression. Dysbiosis of the gut microbiome leads to increased gut permeability, allowing endotoxins (*e.g.,* LPS) to enter the portal circulation and activate KCs and other immune cells in the liver, further promoting inflammation and MASH progression.^156^^,^^157^

### 4.2 FXR and GPBAR1 ligands in MASH

BAs are increasingly recognized as key regulators of hepatic immunity, beyond their classical roles in digestion and metabolism. The receptors FXR and GPBAR1 are pivotal in mediating immune responses within the liver.

Several FXR and GPBAR1 agonists are currently under investigation for their potential to treat MASH.^158^ Based on their selectivity, these agonists can be classified into three main categories: GPBAR1-selective agonists, FXR-selective agonists, and dual GPBAR1/FXR agonists.^55^ Additionally, FXR antagonists are being evaluated for their therapeutic potential.^157^, ^158^, ^159^, ^160^ From a chemical perspective, these agents can be further divided into steroidal (BA and non-BA derivatives) and non-steroidal compounds.^77^^,^^161^

#### 4.2.1 GPBAR1 agonists

Although GPBAR1-deficient mice do not spontaneously develop metabolic disorders, activation of this receptor in preclinical models has yielded promising results, particularly in the context of MASH and metabolic syndrome.^162^

Activation of GPBAR1 in mice fed a high-fat diet by 7β-dihydroxy-5β-cholan-24-ol (BAR501), a selective GPBAR1 agonist, has been shown to improve vascular function, reduce the progression of atherosclerosis, and significantly decrease hepatic fat accumulation.^147^^,^^163^ These effects are particularly noteworthy as they suggest that GPBAR1 agonists could be beneficial in managing the cardiovascular and hepatic complications associated with MASH.^48^^,^^163^, ^164^, ^165^, ^166^ Other GPBAR1 are INT777 and INT767 (a dual FXR and GPBAR1 agonist), both developed by Intercept Pharmaceuticals Inc.^164^ These agents are no longer being developed, as Intercept has abandoned its program of drug discovery in MASLD in 2023 after U.S. FDA rejected approval of OCA in MASLD.

In addition to synthetic agonists, several natural compounds have been identified as GPBAR1 ligands, including oleanolic acid, betulinic acid, and ursolic acid. These naturally occurring substances have shown potential in modulating GPBAR1 activity, although their clinical applicability remains to be fully explored.^161^ Despite the promising preclinical data, there are currently no GPBAR1 agonists in advanced clinical trials for MASH, highlighting an area of unmet medical need and potential therapeutic opportunity.

#### 4.2.2 FXR agonists

FXR activation is essential for maintaining BA homeostasis, regulating lipid and glucose metabolism, and modulating inflammatory responses.^72^^,^^167^, ^168^, ^169^ As a result, FXR agonists have garnered significant attention as potential therapeutic agents for MASH, with several compounds currently in various stages of development.

The most advanced FXR agonist is OCA, a semi-synthetic derivative of CDCA originally developed as INT747.^69^^,^^170^^,^^171^ OCA was approved in 2016 as a second-line treatment for patients with PBC who were resistant to UDCA and has since been extensively studied in MASH.^172^ The Phase 3 REGENERATE trial demonstrated that OCA could significantly improve liver fibrosis in patients with MASH, with 23.1% of treated patients showing a ≥1 stage improvement in fibrosis without worsening MASH, compared to 11.9% in the placebo group. However, the treatment was associated with side effects such as pruritus, occurring in over half of the patients, as well as increases in low-density lipoprotein cholesterol, gallstones, and acute cholecystitis.^173^ Since then, in June 2023, the U.S. FDA rejected Intercept Pharmaceutical’s second bid for approval of OCA for the treatment of MASH with stage 2 or 3 fibrosis due to the severity of side effects (https://www.medscape.com/viewarticle/993603?form=fpf). More recently, the European Medicines Agency recommended revoking the conditional marketing authorization for OCA in the European Union (https://www.ema.europa.eu/en/news/ema-recommends-revoking-conditional-marketing-authorisation-ocaliva), which will lead to market withdraw of this agent.

Other FXR agonists under investigation include EDP-305, a non-BA steroidal FXR agonist developed by Enanta Pharmaceuticals Inc.^158^^,^^174^ EDP-305 has shown promise in early-phase clinical trials, with evidence of FXR activation, as indicated by increased levels of FGF19 and reduced BA synthesis. Despite its potential, EDP-305 has also been associated with adverse effects such as pruritus and alterations in lipid profiles, which may limit its utility in certain patient populations.^174^

Non-steroidal FXR agonists, such as cilofexor and tropifexor,^174^ represent a newer generation of FXR-targeting compounds with the potential for improved safety and efficacy profiles.^175^ Cilofexor, developed by Gilead Sciences, has shown significant reductions in liver fat content in MASH patients during Phase 2 trials, although its efficacy was moderated by dose-dependent pruritus and modest changes in liver enzymes.^176^ Tropifexor, developed by Novartis, has demonstrated similar efficacy in reducing liver fat and ALT levels, with a generally favorable safety profile, making it a promising candidate for further clinical evaluation.^158^^,^^174^ These non-steroidal agonists are particularly attractive due to their potential to offer a more targeted therapeutic approach with fewer side effects.^174^

#### 4.2.3 Dual GPBAR1 and FXR agonists

Dual agonists that target both GPBAR1 and FXR represent a novel and exciting approach to the treatment of MASH. The rationale behind dual agonism is to leverage the complementary roles of these receptors in regulating BA metabolism, inflammation, and fibrosis, potentially offering a more comprehensive therapeutic strategy.^174^

Studies on mice with dual knockout of GPBAR1 and FXR have revealed significant disruptions in BA homeostasis and an increased susceptibility to liver fibrosis, underscoring the importance of these receptors in maintaining liver health.^177^^,^^178^ 7α-dihydroxy-24-nor-5β-cholan-23-ol (BAR502), a dual GPBAR1/FXR agonist developed by BAR Pharmaceuticals, has shown significant efficacy in preclinical models of MASH. This compound, which exhibits a slight preference for GPBAR1, has been demonstrated to reduce liver fat accumulation, improve metabolic profiles, and attenuate fibrosis in animal studies, suggesting its potential as a therapeutic agent for MASH.^161^^,^^179^, ^180^, ^181^

Another dual agonist, INT767, also targets both GPBAR1 and FXR. This compound not only modulates BA metabolism but also exerts anti-inflammatory and antifibrotic effects, but its development has been abandoned by Intercept Pharmaceuticals Inc.^182^^,^^183^ Although still in the early stages of clinical development, BAR502 holds promise in the treatment of MASH, with the potential to address multiple aspects of the disease simultaneously.^48^^,^^147^^,^^184^

### 4.3 BARs in PBC and PSC

Immune-mediated hepatic disorders such as PBC and PSC are characterized by chronic cholestasis, inflammation, and progressive fibrosis, which can lead to cirrhosis and liver failure.^73^ BARs, particularly FXR and GPBAR1, play crucial roles in modulating these immune responses and maintaining hepatic homeostasis.^185^

PBC is a chronic, progressive liver disease characterized by the immune-mediated destruction of intrahepatic bile ducts, leading to cholestasis and liver damage. The disease is marked by high titers of circulating anti-mitochondrial antibodies, particularly those directed against the estradiol subunit of the pyruvate dehydrogenase complex. These antibodies, along with autoreactive T cells, contribute to the chronic inflammation and bile duct damage observed in PBC.^186^^,^^187^

PSC is a chronic cholestatic liver disease characterized by inflammation and fibrosis of both intrahepatic and extrahepatic bile ducts, leading to bile duct strictures and eventually cirrhosis. PSC is often associated with other autoimmune conditions, particularly IBD, and presents a distinct immune-mediated pathogenesis involving both innate and adaptive immune responses.^188^^,^^189^

#### 4.3.1 Role of GPBAR1 in PBC and PSC

Activation of GPBAR1 in cholangiocytes enhances the secretion of bicarbonate, which forms a protective “bicarbonate umbrella” that safeguards the biliary epithelium from BA toxicity. Additionally, GPBAR1 activation leads to the upregulation of junctional adhesion molecule A (JAM-A), which strengthens the epithelial barrier function, reducing bile leakage and maintaining the integrity of the biliary tree.^190^^,^^191^

In KCs, GPBAR1 activation exerts anti-inflammatory effects by inhibiting the NF-κB signaling pathway, thereby reducing the production of proinflammatory cytokines. This mechanism plays a crucial role in modulating the immune response and protecting the liver from chronic inflammation.^45^^,^^192^^,^^193^ Although specific GPBAR1 agonists have not yet been developed for clinical use in PBC, GPBAR1 remains a promising therapeutic target due to its potential to modulate immune responses and protect against bile duct damage.

GPBAR1 plays a protective role in PSC by modulating inflammatory responses and enhancing bile duct integrity.^194^ Studies have shown that the expression of GPBAR1 is significantly reduced in cholangiocytes from PSC patients. However, treatment with UDCA or norUDCA has been shown to counteract this downregulation, suggesting a therapeutic role for GPBAR1 activation in managing PSC.^53^

Moreover, recent genetic studies have identified mutations in the GPBAR1 gene in PSC patients, with certain variants leading to impaired receptor function.^195^^,^^196^ These findings underscore the potential of GPBAR1 as a therapeutic target in PSC, although clinical trials are still needed to explore the efficacy of GPBAR1 agonists in this context.

#### 4.3.2 Role of FXR in PBC and PSC

By inhibiting the NF-κB pathway, FXR reduces the production of proinflammatory cytokines and limits the activation of the immune system, thereby mitigating the chronic inflammation associated with PBC.^197^, ^198^, ^199^

FXR also inhibits the NLRP3 inflammasome in cholangiocytes, further reducing inflammation and preventing bile duct damage.^71^ OCA, a potent FXR agonist, has been approved as a second-line treatment for PBC, particularly in patients with an inadequate response to UDCA.^172^ Clinical trials have demonstrated that OCA improves liver function and prolongs transplant-free survival, although its use is associated with side effects such as pruritus and, in rare cases, liver decompensation.^200^ Despite these challenges, FXR activation remains a central therapeutic strategy in the management of PBC.

FXR activation in PSC is associated with reduced inflammation and fibrosis, similar to its effects in PBC. FXR inhibits the activation of the NLRP3 inflammasome in cholangiocytes, reducing the inflammatory response and protecting the bile ducts from further damage.^71^ While OCA, an FXR agonist, has shown promise in clinical trials, its use in PSC is not as well established as in PBC.^38^ Further research is needed to determine the full therapeutic potential of FXR activation in PSC, particularly in combination with other therapies.^73^

#### 4.3.3 Role of RORγt in PBC and PSC

Increased Th17 cell activity and elevated IL-17 levels have been observed in patients with PBC, suggesting that RORγt-driven Th17 cells contribute to the inflammatory processes underlying the disease, indicating a mechanistic role for RORγt in the pathogenesis of PBC.^201^^,^^202^ RORγt is essential for the differentiation of Th17 cells, which have been shown to play a proinflammatory role in autoimmune diseases, including PBC. Elevated levels of IL-17 are associated with bile duct injury and liver fibrosis in patients with PBC, highlighting the contribution of RORγt-mediated pathways to disease progression.^203^ Additionally, in PBC, there is often a dysregulation between Th17 cells and Tregs, resulting in a skewing towards a proinflammatory Th17 response. RORγt, by promoting Th17 cell differentiation, may tip this balance, leading to enhanced autoimmune responses against the bile ducts. Finally, RORγt-driven Th17 cells interact with other immune cells, such as macrophages and B cells, contributing to the overall inflammatory milieu in PBC. This contributes to the chronic immune-mediated destruction of bile ducts. Clinical and experimental data support the idea that targeting the Th17/RORγt axis could be beneficial in PBC, and therapies aimed at inhibiting IL-17 or modulating Th17 cell responses are currently being explored as potential treatments for PBC and other autoimmune liver diseases.^204^

Similarly to PBC, in PSC, there is significant immune dysregulation, particularly involving the interaction between the gut and liver. The imbalance between proinflammatory Th17 cells and Treg is thought to drive chronic inflammation in PSC, and RORγt plays a central role in promoting Th17 cell differentiation.^205^ There is evidence that Th17 cells and IL-17 levels in the liver and blood increase in PSC patients, suggesting a role of Th17 cells and RORγt in sustaining bile duct inflammation and fibrosis.^206^ Emerging evidence also points to a critical role of gut microbiota in PSC pathogenesis.^207^ RORγt influences intestinal immune responses, particularly through Th17 cells, which interact with the gut microbiota. An intestinal dysbiosis is common in PSC patients and may lead to enhanced Th17 activity and, consequently, increased liver inflammation through the gut-liver axis, making RORγt a potential target in PSC.^139^^,^^208^

## 5 Conclusions

In addition to their role in lipid absorption, BAs function as signaling molecules that interact with nuclear and membrane-bound receptors, modulating intestinal and liver immunity. The two main BARs, FXR and GPBAR1, along with RORγT, VDR, and PXR, play integral roles in regulating liver immune responses. FXR and GPBAR1 are robustly expressed in cells of innate immunity, monocytes/macrophages, and NK cells. FXR, the primary BA sensor, is activated by primary BAs and modulates liver innate immunity by suppressing proinflammatory pathways, including NF-κB signaling, and by inducing anti-inflammatory cytokines like IL-1 receptor antagonists. FXR activation inhibits liver fibrosis by reducing the activation of HSCs and maintaining macrophage balance. GPBAR1, activated by secondary BAs, promotes the release of anti-inflammatory cytokines (*e.g.,* IL-10) and enhances macrophage polarization towards an anti-inflammatory M2 phenotype. In contrast to FXR and GPBAR1, RORγt is restricted to T cells and ILC3 and is critical for the differentiation of Th17 cells. BAs, specifically 3-oxoLCA (3-oxo-12α-hydroxy-5β-cholanoic acid) and isoalloLCA, have been identified as potent modulators of RORγt. Both isolalloLCA and 3-oxoLCA function as RORγt, inhibitors, reducing the differentiation of Th17 cells and potentially offering therapeutic implications for autoimmune and inflammatory disorders, including MASLD, PBC, and PSC. LCA and its derivative 3-oxoLCA function as dual VDR agonists and RORγt antagonists, establishing a functional role for these agents in regulating both innate and adaptive immunity. The discovery of novel families of secondary BAs, such as the amidated BAs, has revealed the astonishing chemical diversity of the critical component of the postbiota, opening new opportunities for drug discovery.

## Authors’ contributions

**Stefano Fiorucci:** Writing – review & editing, Writing – original draft, Conceptualization. **Silvia Marchianò:** Writing – review & editing. **Eleonora Distrutti:** Writing – review & editing. **Michele Biagioli:** Writing – review & editing, Writing – original draft, Conceptualization. All authors have read and agreed to the published version of the manuscript.

## Declaration of competing interest

Stefano Fiorucci is an associate editor for *Liver Research* and was not involved in the editorial review or the decision to publish this article. All authors declare that there are no competing interests.
